# Taxon- and Site-Specific Melatonin Catabolism

**DOI:** 10.3390/molecules22112015

**Published:** 2017-11-21

**Authors:** Rüdiger Hardeland

**Affiliations:** Johann Friedrich Blumenbach Institute of Zoology and Anthropology, University of Göttingen, Bürgerstr 50, D-37073 Göttingen, Germany; rhardel@gwdg.de; Tel.: +49-551-395415

**Keywords:** 5-methoxytryptamine, CNS, dinoflagellates, indole metabolism, kynuramines, plants, yeast

## Abstract

Melatonin is catabolized both enzymatically and nonenzymatically. Nonenzymatic processes mediated by free radicals, singlet oxygen, other reactive intermediates such as HOCl and peroxynitrite, or pseudoenzymatic mechanisms are not species- or tissue-specific, but vary considerably in their extent. Higher rates of nonenzymatic melatonin metabolism can be expected upon UV exposure, e.g., in plants and in the human skin. Additionally, melatonin is more strongly nonenzymatically degraded at sites of inflammation. Typical products are several hydroxylated derivatives of melatonin and *N*^1^-acetyl-*N*^2^-formyl-5-methoxykynuramine (AFMK). Most of these products are also formed by enzymatic catalysis. Considerable taxon- and site-specific differences are observed in the main enzymatic routes of catabolism. Formation of 6-hydroxymelatonin by cytochrome P_450_ subforms are prevailing in vertebrates, predominantly in the liver, but also in the brain. In pineal gland and non-mammalian retina, deacetylation to 5-methoxytryptamine (5-MT) plays a certain role. This pathway is quantitatively prevalent in dinoflagellates, in which 5-MT induces cyst formation and is further converted to 5-methoxyindole-3-acetic acid, an end product released to the water. In plants, the major route is catalyzed by melatonin 2-hydroxylase, whose product is tautomerized to 3-acetamidoethyl-3-hydroxy-5-methoxyindolin-2-one (AMIO), which exceeds the levels of melatonin. Formation and properties of various secondary products are discussed.

## 1. Introduction

While melatonin biosynthesis is frequently studied and considered to be highly relevant, the awareness of melatonin catabolism is largely restricted to a very few compounds, among which some of them are often thought to be more or less irrelevant because they are mostly found in low quantities. The relatively fast hepatic catabolism by cytochrome P_450_ subforms that preferably leads to 6-hydroxymelatonin is known to limit the presence of melatonin in the blood. Therefore, this process is often believed to represent the only route of biological significance. However, at a closer look, such a view turns out to be too much centered on the circulation and on mammals. The disregard of melatonin catabolism in other organs can be misleading, since metabolites other than 6-hydroxymelatonin may attain relevant concentrations in some tissues and also in the cerebrospinal fluid (CSF), as will be discussed in this article. Moreover, conditions can exist under which another catabolic route can become more important, as has been found to occur under the influence of inflammation [1,2]. Generally, it seems important to distinguish between the different roles of melatonin within an organism, roles that exceed that of a hormone in the classic definition [3] as being released from a specific gland and distributed via the circulation. Melatonin is known to be synthesized in numerous organs and cells, and the quantities of extrapineal melatonin are by orders of magnitude higher than those in the pineal gland and in the circulation [4,5]. The role of 6-hydroxymelatonin is closely associated with melatonin’s function of a short-lived chronobiological signaling factor that is rapidly eliminated by conversion to an easily excretable compound. 6-Hydroxylation is the prerequisite of conjugation, mainly by sulfation, which leads to the urinary metabolite 6-sulfatoxymelatonin. These requirements are not necessarily valid for many other tissues that produce melatonin in high quantities, but often release it only in low amounts [4,6]. The fate of extrapineal melatonin in tissues is often incompletely understood.

Another need related to considering other routes of melatonin catabolism follows from the discovery of this compound in almost all taxa tested, including bacteria, various phyla of eukaryotic unicells, plants, fungi and invertebrate animals [7,8,9]. As will be outlined in this article, considerable differences exist concerning the quantitative prevalence of specific catabolic pathways between major taxa. The differences will be also discussed in terms of the properties of the respective major metabolites. These properties can be decisive for understanding the biological meaning of the pathways in ecologically different groups of organisms. Moreover, these considerations will not only be restricted to enzymatic mechanisms, but also comprise nonenzymatic reactions based on reactive oxygen and nitrogen species (ROS, RNS) and, in particular, photochemical reactions that are of importance at sites exposed to UV light.

## 2. CYP-Based Metabolism

**Figure 1:**
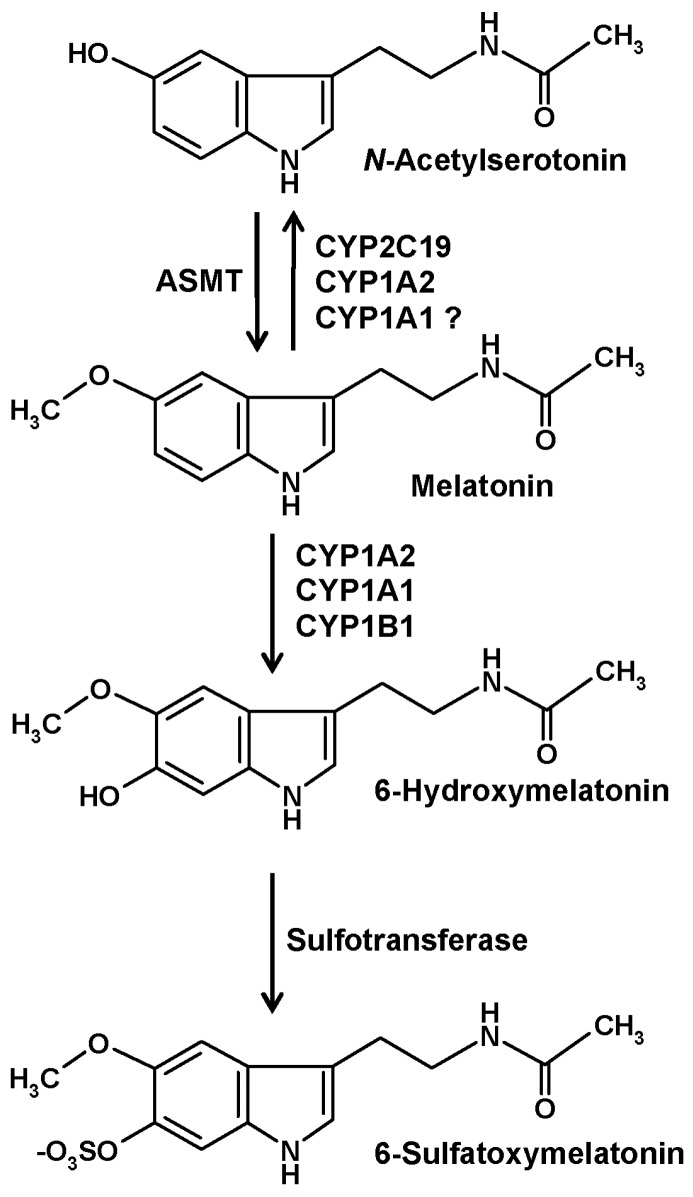
The main catabolic route of circulating melatonin in vertebrates. Abbreviations: ASMT, *N*-acetylserotonin *O*-methyltransferase (formerly known as hydroxyindole *O*-methyltransferase, HIOMT); CYP, cytochrome P_450_. Hydroxylation and demethylation reactions by CYP isoforms, as indicated, are not only known from liver, but also from the brain [1]. Nonenzymatic formation of 6-hydroxymelatonin by free radicals is also chemically possible, but of minor importance relative to the CYP-dependent pathway.

CYPs are the major enzymes of vertebrate melatonin metabolism, especially in the liver, but also in other tissues. In quantitative terms, hydroxylation by hepatic CYP1A2 to 6-hydroxymelatonin represents the prevailing mechanism, but other isoforms, in particular, CYP1A1 and the nonhepatic CYP1B1, to a smaller extent also CYP2C19, can catalyze the same reaction [10] (Figure 1). Hydroxylation at ring atom 6 is required for conjugation, mostly by sulfation, to a minor amount by glucuronidation. In either case, the hydrophilic conjugate is easily excreted. Urinary levels of 6-sulfatoxymelatonin are usually regarded as an indirect, temporally integrating measure of melatonin production by the pineal gland [11].

An alternate catabolic route catalyzed by CYP isoforms is that of dealkylation, a general property of many CYPs. However, this pathway is usually regarded to be of minor importance for melatonin. The formation of *N*-acetylserotonin by demethylation (Figure 1) leads to the difficulty that this compound is also the precursor of melatonin, two roles that cannot be easily distinguished under experimental settings. Concerning subforms that accept melatonin as a substrate, demethylation reactions are known for CYP2C19 and CYP1A2 and may, perhaps, also be possible with CYP1A1 [1]. A third reaction type that can be catalyzed by CYP1A2 using other substrates, epoxide formation [12], has not been directly studied with melatonin and may appear to be rather unlikely. An epoxide is easily hydrolyzed to a dihydroxylated compound. Epoxidation would only be, theoretically, possible in two places at the indolic moiety of melatonin, at ring atoms 2 and 3 or at ring atoms 6 and 7. No report exists for a formation of 6,7-dihydroxymelatonin. Hydrolysis of a 2,3-epoxide would lead to a dihydroxylated product that would immediately turn into the respective keto tautomer, 3-acetamidoethyl-3-hydroxy-5-methoxyindolin-2-one. This compound, which is sometimes misleadingly called 2,3-dihydroxymelatonin, is known as a melatonin metabolite, but its formation has been described to be catalyzed by cytochrome c rather than CYPs [13]. This product easily undergoes a rearrangement to a kynuric metabolite, as will be discussed in another section.

Several of the melatonin-catabolizing CYPs are also expressed in nonhepatic tissues. Although their quantitative contribution to circulating 6-hydroxymelatonin levels and to urinary 6-sulfatoxymelatonin is only minor, local metabolism in other organs may not be entirely irrelevant. This is particularly valid for the central nervous system (CNS). In the brain, the expression of CYP1A2 [14,15,16], CYP1A1 [14,16], CYP1B1 [10,16,17], and CYP2C19 [18,19,20] has been demonstrated. The contribution of the CYP2C19 to melatonin demethylation and that of CYP1B1 to 6-hydroxylation in the brain may be higher than in other tissues [1]. Interestingly, 6-hydroxymelatonin is also sulfated in the CNS [1,21], although the role of 6-sulfatoxymelatonin in the brain as well as its elimination from there has remained entirely unclear [1].

Although CYP-mediated 6-hydroxylation represents a major route of vertebrate melatonin catabolism, this does not seem to play any relevant role in other organisms. At least, no reports on relevant quantities of 6-hydroxy- or 6-sulfatoxymelatonin exist to date for invertebrate animals, fungi, plants and algae. This difference is particularly remarkable in the case of plants, which express high numbers of different CYP isoforms that might be suspected to also catalyze 6-hydroxylation and *O*-demethylation of melatonin [22]. Instead, another hydroxylation reaction at ring atom 2, which will be described in the subsequent section, has turned out to be the prevailing catabolic route and to be independent of CYPs [22]. CYP-independent hydroxylations of melatonin are also possible by nonenzymatic processes, e.g., by interaction with free radicals, as will be discussed next.

## 3. Other Hydroxylation Mechanisms

Hydroxylations are possible by enzymatic and nonenzymatic reactions. From a fundamental point of view, nonenzymatic hydroxylation by free radicals should be possible in any aerobic species, in any cell type and also in the extracellular space. However, this does not imply by any means that the rates of these reactions are always more or less the same. In fact, the quantitative relevance of these reactions can strongly vary, depending on free radical-generating processes that may be strongly increased, in vertebrates, e.g., by inflammation or, in many organisms, at sites exposed to environmental stress, such as UV radiation, in phototroph species even by visible light that causes free radical formation in the photosystems.

Hydroxylation by interaction with free radicals, in particular, via consecutive reactions with two hydroxyl radicals (•OH), are principally possible at any unsubstituted C-atom of the indole moiety [23]. However, not all of the possible products are of equal biological or medical interest. Nonenzymatically formed 6-hydroxymelatonin represents only a very minor fraction relative to the CYP-generated quantities. 4-Hydroxymelatonin has recently attracted some attention, as it was reported to be an excellent radical scavenger with potency for eliminating peroxyl radicals higher than that of melatonin [24]. Previously, 4-hydroxymelatonin was detected as a metabolite in keratinocytes, in which its formation was strongly enhanced by UV B [25].

**Figure 2:**
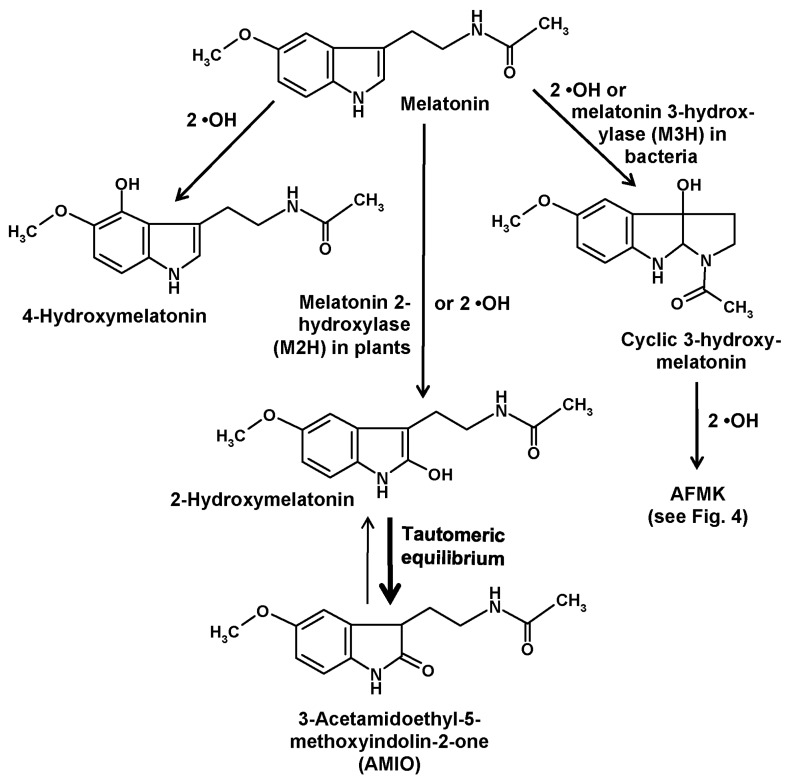
Hydroxylation reactions at ring atoms 2, 3, and 4 of melatonin. Abbreviation: AFMK, *N*^1^-acetyl-*N*^2^-formyl-5-methoxykynuramine.

Another compound of relevance is formed by hydroxylation at ring atom 3. This change causes an immediate intramolecular rearrangement that leads to the formation of a third ring, to give a metabolite denominated as cyclic 3-hydroxymelatonin [26,27] (Figure 2). This had first been discovered as a combination of hydrogen abstraction by a •OH and addition of a second one [26,27]. Its formation was increased by administration of melatonin [27] and, strongly, by exposure to ionizing radiation [26]. Cyclic 3-hydroxymelatonin was shown to also be a potent free radical scavenger [28] and to be converted by two •OH to another key metabolite of melatonin, *N*^1^-acetyl-*N*^2^-formyl-5-methoxykynuramine (AFMK) [29], which will be discussed in detail in a following section. Until recently, no enzymatic formation of cyclic 3-hydroxymelatonin was known. This gap has now been closed, but to date this information is restricted to bacteria. In *Escherichia coli*, three enzymes belonging to the 2-oxoglutarate-dependent dioxygenase (2-ODD) superfamily, 2-ODD 11 (most active form), 2-ODD 26, and 2-ODD 33, were shown to be capable of converting melatonin into cyclic 3-hydroxymelatonin [30]. Rice plants carrying such a melatonin 3-hydroxylase (M3H) transgene produced the expected metabolite and also its secondary product, AFMK. Exposure to cadmium, which is known to increase melatonin levels in rice also increased the apparent M3H activity [30]. However, product specificities of the M3H subforms 2-ODD 11 and 2-ODD 33 were somewhat incomplete, since these isoenzymes hydroxylated melatonin also at ring atom 2, though at comparably low rates [30]. Actually, the demonstration of enzymatic 3-hydroxylation of melatonin has only validity for *E. coli*, but may be soon extended to other bacteria. As melatonin is present in bacteria [8] and has been also detected in *E. coli* [31], a functional role of M3H enzymes may exist, but this remains to be demonstrated. Concerning other organisms, the possibility of enzymatic 3-hydroxylation has not yet been tested. With regard to low basal quantities of cyclic 3-hydroxymelatonin, this may be rather unlikely in vertebrates, but many other major taxa have not been investigated in this regard.

Hydroxylation of melatonin at ring atom 2 has also been shown to exist both enzymatically and nonenzymatically. As with comparable hydroxylations at other ring atoms, this is also possible by consecutive interactions with two •OH [32,33]. These findings are in line with the observation that 2-hydroxymelatonin is a cutaneous photoproduct that is strongly increased by UV B, although certain amounts of this compound were also detected in non-irradiated keratinocytes [25]. Moreover, formation of 2-hydroxymelatonin was reported to occur under the influence of HOCl [34] and, later, of taurine chloramine [35], a reactive intermediate generated by activated neutrophils from HOCl and taurine. Therefore, activation of myeloperoxidase during local inflammatory responses seems to cause nonenzymatic 2-hydroxylation of melatonin.

2-Hydroxylation of melatonin by side reactions of enzymes with different main functions has been occasionally communicated. For instance, 2-hydroxymelatonin has been found to represent an intermediate metabolite formed by cytochrome c in an AFMK-generating pathway [13], a result that might explain the formation of 2-hydroxymelatonin in non-irradiated keratinocytes. Horseradish peroxidase was also reported to hydroxylate melatonin at ring atom 2, although substantial rates were only observed at pH 5.5 [36]. Additionally, a dimer of 2-hydroxymelatonin was detected under these conditions [36].

In plants, a considerably higher physiological relevance exists for 2-hydroxylation by another 2-ODD that has turned out to be rather specific, exhibits relatively high activities and is responsible for the prevailing melatonin catabolizing pathway in, at least, angiosperms, perhaps also in all plants (but not in all phototrophs). This enzyme has been denominated as melatonin 2-hydroxylase (M2H) and exists, in species studied in this regard, in multiple subforms. In rice, three subforms were present in the cytosol, whereas another one was expressed in chloroplasts [37]. Studies on cloned M2H unequivocally showed that, in plants, the 2-hydroxymelatonin pathway was predominantly an enzymatic one [38]. In quantitative terms, the amounts of metabolites formed in this route was remarkable and, for most experts, surprising. The products attained levels by orders of magnitude higher (average 368-fold) than those of the parent compound melatonin, at least, in several plants studied [39].

Of course, this unexpected proportion requires explanation and raises the question on its biological significance. First, it is important to be aware of the tautomery of a 2-hydroxylated indolic compound (Figure 2). In most literature, authors use the term “2-hydroxymelatonin”, when they mean, in fact, another compound, namely, its keto tautomer, 2-acetamidoethyl-5-methoxyindolin-2-one. This indolinone [33,40], sometimes also referred to as an oxindole, is known since long to be the prevailing tautomer relative to the enolic hydroxyindole [32] and has been recently reported to represent almost 100% of the keto/enol mixture [24]. Using the expression “2-hydroxymelatonin” for its indolinone tautomer is highly misleading, since it inappropriately seems to indicate properties of this molecule reminiscent of those known for melatonin. To discriminate the indolinone from melatonin and other hydroxylated melatonin derivatives, especially in terms of properties, it would be preferable to apply a more correct terminology. Instead of the relatively long chemical name, an abbreviation may be used, such as “AMIO” (Figure 2), as had also been done and is now customary with other melatonin metabolites such as AFMK.

The differences in properties mainly concern two aspects, those of reactivity and of lipophilicity. Contrary to melatonin and several of its hydroxylated derivatives, such as cyclic 3-hydroxymelatonin, 4-hydroxy- and 6-hydroxymelatonin, the metabolite AMIO is much less reactive and a relatively poor direct antioxidant [24]. This means that AMIO will not be easily removed by oxidants, but the low reactivity may also disfavor a rapid enzymatic elimination. The removal of AMIO from biological material remains to be clarified. One possibility was detected in a dermatological context [13], as which will be discussed below, but the quantitative relevance of this route in plants remains questionable, especially with regard to the extremely high amounts found there. Moreover, the change from a hydroxyindole to an indolinone leads to a substantial increase in lipophilicity, which can be easily seen in chromatograms. Therefore, it would be of interest to know how AMIO is distributed in plant cells that contain high amounts of this metabolite. In particular, the possibility should be tested that AMIO might be trapped in lipids, such as compartments with high amount of membranes, e.g., chloroplasts, or in lipid droplets. The recently started and actually progressing studies on the functional role of AMIO may shed light on this problem. A study testing MAP kinase activation in the context of pathogen resistance revealed activation of MAPK3 and MAPK6 by AMIO, however, to a smaller extent as observed with melatonin [41]. At least, one would assume from this report that AMIO should be sufficiently available in the aqueous phase to act on other proteins. Another investigation showed that AMIO contributed to cold and drought resistance, upregulated respective transcription factors, caused increases in the osmoprotectant amino acid proline, and supported mitochondrial integrity under cold and drought stress [42]. These initial findings indicate that AMIO is, in fact, a bioactive compound derived from melatonin and contributes to overall effects of the parent compound.

## 4. The Deacetylation Pathway

**Figure 3:**
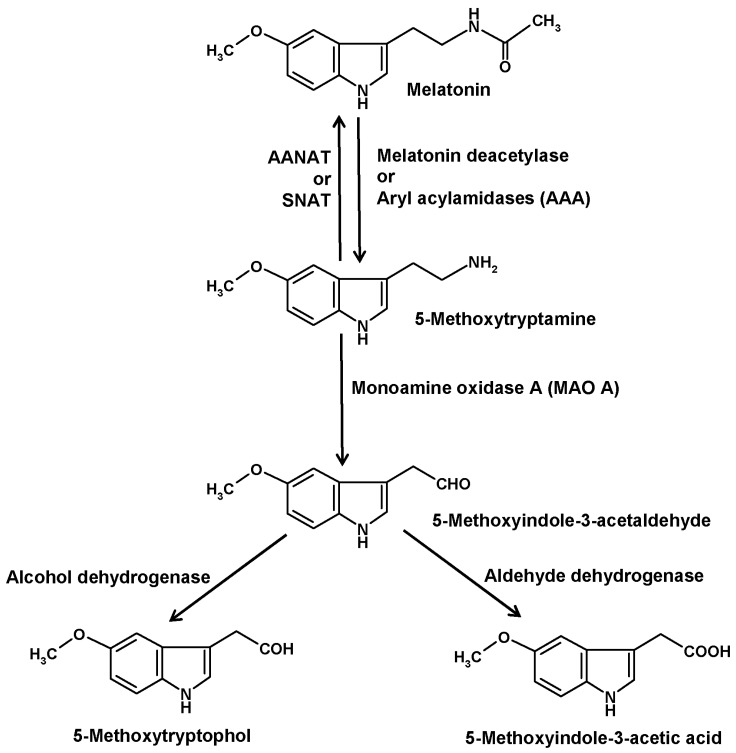
Role of 5-methoxytryptamine in melatonin metabolism.

Another catabolic route is initiated by deacetylation of melatonin to 5-methoxytryptamine (5-MT) (Figure 3). The existence of this pathway is insofar remarkable as 5-MT can also serve as a precursor of melatonin in the alternate pathway of melatonin synthesis that seems to prevail in various organisms outside the animals [43]. This duality in the role of 5-MT was particularly evident in *Saccharomyces*. When starved yeast cells were supplied with exogenous melatonin, a large fraction was converted to 5-MT, but when these cells received 5-MT, they formed melatonin [44]. Substantial amounts of 5-MT were also obtained when cells were supplemented with *N*-acetylserotonin [45].

Deacetylation of melatonin has been described for several enzymes. In older literature, such enzymes were generally referred to as aryl acylamidases (AAAs). Moreover, AAA side activities were described for acetylcholinesterase [46], also under the name of AAA-2 [47], and butyrylcholinesterase [48,49,50], but their roles as melatonin-converting enzymes seem to be low or irrelevant. Other AAAs were detected in liver [51,52], brain [47] and pineal gland [53]. However, the brain enzyme did not catabolize melatonin, whereas this was demonstrated for the hepatic isoform [51,52] and, indirectly, for that from the pineal [53]. 5-MT was also shown to appear in the blood after injection of melatonin [52]. In eyes and pineal glands of the European mole (*Talpa europaea*) [54] and in pineals of Syrian hamsters [55,56], 5-MT had been measured. However, safe determinations required the inhibition of 5-MT catabolism by blocking MAO A [57]. Under such conditions, a circadian rhythm of 5-MT was detected in the hamster pineals [55,56]. This rhythm strongly differed from that of melatonin and was rather reminiscent of the serotonin rhythm. As 5-MT exerted several biological effects, especially in the reproductive system, it was for a while discussed as another pineal hormone [58].

Our understanding of 5-MT formation from melatonin, which had been also observed in the eyes of *Xenopus laevis* [59], was considerably improved by attributing it to a specific melatonin deacetylase [60]. This was shown to also apply to findings on retina and/or pineal gland in other nonmammalian species, such as the teleost fish *Carassius auratus*, the lizards *Anolis carolinensis* and *Sceloporus jarovi*, and the chicken, *Gallus domesticus* [61,62]. In *Xenopus*, melatonin deacetylase was also detected in the skin [61]. Recently, high amounts of 5-MT that exceed those of melatonin by an order of magnitude have been reported for the human skin [63], but it may be possible that this finding reflects the alternate pathway of melatonin synthesis rather than formation by deacetylation.

The difference between AAA and melatonin deacetylase should be mainly seen from the viewpoint of substrate specificity. Melatonin deacetylase is relatively selective for melatonin, whereas AAAs have much lower substrate specificity and cleave many other aryl acylamides, too, including acetanilide as well as its nitrated and chlorinated derivatives. In search of an enzymes degrading paracetamol (=*p*-acetaminophenol = acetaminophen), a bacterial AAA was discovered [64]. This type of enzymes seems to be phylogenetically old, but is has not been tested whether a bacterial AAA would accept melatonin as a substrate. Although AAAs exist in plants [65] and melatonin is also present, sometimes at strongly elevated levels [22,66], no substrate relationship has become known and may not have been studied.

However, a substantial role of melatonin deacetylation became evident in another phototrophic organism, the marine dinoflagellate *Lingulodinium polyedrum* (syn. *Gonyaulax polyedra*). Although many but not all dinoflagellates are phototrophs, these organisms are not phylogenetically related to plants, but rather belong to the clade of Alveolata. In many dinoflagellates, 5-MT exerts profound effects of ecophysiological relevance. In various species such as *L. polyedrum* [67,68] and others from related genera [69,70], 5-MT is a strong inducer of asexual cysts, i.e., of resting stages that allow the survival of cells under adverse conditions. In several other dinoflagellate species, cells responded to 5-MT by immobilization, but not formation of protective cyst pellicles [70]. In bioluminescent dinoflagellates, cyst formation was preceded and accompanied by very strong rises in light emission, effects that were mediated by a cytoplasmic acidification that activates the light-producing system in the bioluminescent microsources and also favors the transition to the resting state [71,72].

Melatonin deacetylation is catalyzed in *L. polyedrum* by an enzyme that has been classified as AAA rather than melatonin deacetylase. Two reasons were decisive. First, the enzyme has a broader specificity and accepts, e.g., *N*-chloroacetyl-l-tryptophan as a substrate [73], a property that had been earlier shown to be favorable for developing colorimetric AAA assays [74]. Second, the *Lingulodinium* AAA was not inhibited by eserine [75], a blocker of melatonin deacetylase from *Xenopus* [60,61]. As eserine is also an inhibitor of acetylcholinesterase including its AAA side activity, the enzyme from the dinoflagellate is also different from this enzyme, which would be also in line with a lack of demonstrable acetylcholinesterase activity in this species [75].

In *Lingulodinium*, the relationship between melatonin and 5-MT is a rather unusual one. In this organism, which also exhibits a nocturnally peaking circadian rhythm of melatonin [76], an exceptionally strong response exists to moderate decreases in temperature. A step from the rearing temperature of 20 °C to 15 °C leads to rises in melatonin from about 1 µM at circadian maximum, or much less at the minimum, to concentrations almost approaching the mM level [76,77], the strongest temperature effect on melatonin ever described. In the biological context, decreases in temperature of a few degrees occur in the course of vertical migration of these cells, which descend in the evening to deeper water layers for taking up nitrate, whereas they ascend in the morning for starting photosynthesis.

However, the very high levels of melatonin are only briefly maintained, because the elevated levels induce AAA, which relatively soon converts much melatonin and generates high amounts of 5-MT [76,77]. Under these conditions, a decision can be made by cells concerning the advantage of either persisting in an active life state as a motile cell or of transgressing into the resting state of an asexual cyst. If photoperiods are short and, thus, carbon supply is low, this may be in favor of resting as a cyst. If the deep water is very rich in the limiting nutrient nitrate, this may be a good reason for continuing in the active state. Therefore, the cell may integrate these divergent factors. High nitrate was shown to partially block the increase in melatonin and, thus, in the precursor 5-MT [66,78]. As a consequence, the combination of lower temperature, short photoperiod and low nitrate availability leads to cyst-inducing levels of 5-MT, whereas long photoperiods, which are in favor of photosynthesis, and high nitrate suppress this response, even at a moderately lowered temperature [66,78]. In a tropical strain of another dinoflagellate species, *Amphidinium carterae*, collected from the Indonesian sea, the response to temperature was entirely different. In this organism, a higher temperature of 30 °C caused rises in melatonin and 5-MT [66], perhaps, a stress response.

In vertebrates, 5-MT is further catabolized by monoamine oxidase A (MAO A) to 5-methoxyindole-3-acetaldehyde (Figure 3) [33]. In dinoflagellates, a corresponding MAO catalyzed the same reaction, although no pharmacological classification of MAO subtypes exists for this group of organisms. Several MAO inhibitors tested were able, by increasing 5-MT levels, to strongly stimulate bioluminescence and to induce encystment [71,79,80]. In vertebrates as well as in dinoflagellates, 5-methoxyindole-3-acetaldehyde is converted by alcohol dehydrogenase to 5-methoxytryptophol (5-ML) or, alternately, by aldehyde dehydrogenase to 5-methoxyindole-3-acetic acid (5-MIAA) [33,81]. In vertebrates, 5-ML was discussed as a bioactive compound in the CNS and as the source of another potential metabolite, *O*-acetyl-5-hydroxytryptophol, a structural analog of melatonin, in which the aliphatic nitrogen is replaced by an oxygen [81]. However, it should not be overlooked that 5-ML can also be formed by *O*-methylation of the serotonin metabolite 5-hydroxytryptophol or from 5-methoxytryptophan, the latter route being also a possible source of 5-MIAA. In dinoflagellates, 5-ML was typically present in all chromatograms containing melatonin and 5-MT, and was strongly increased under conditions of enhanced melatonin deacetylation [76]. Although 5-ML regularly appeared when high amounts of 5-MT were formed, the major final metabolite turned out to be 5-MIAA [82,83]. Relative to other melatonin metabolites, 5-MIAA has the advantage of being, as an acid, most easily released into the seawater, which has an alkaline pH of 8 or higher that prevents a reuptake. For this reason, 5-MIAA was previously poorly detected in the dinoflagellate cells, but found in substantial quantities in the medium. In the natural environment, 5-MIAA is soon diluted. Under laboratory conditions, no effects of 5-MIAA were observed in dinoflagellates. Whether this metabolite, which is a homolog of indole-3-acetic acid and, thus, possesses properties of an auxin, may have effects in chlorophyceans or aquatic plants, has not been investigated at naturally possible concentrations.

## 5. Formation of Kynuramines and Their Secondary Products

**Figure 4:**
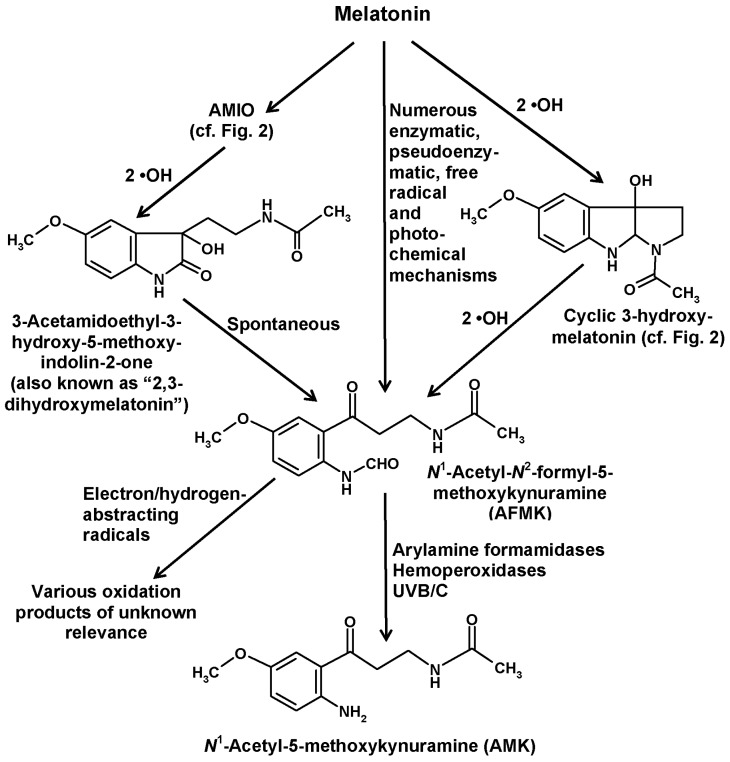
Formation of the kynuric metabolites AFMK and AMK from melatonin. Abbreviation: AMIO, 3-acetamidoethyl-5-methoxyindolin-2-one. For further details of reactions leading to AFMK formation from melatonin see References [29,89,90]. Products from oxidation of AFMK by electron/hydrogen-abstracting radicals have been characterized in Reference [121].

In addition to the indolic routes of melatonin catabolism, a non-indolic pathway also exists, which leads to substituted kynuramines and has been referred to as the kynuric pathway [29]. The major metabolites of this pathway are AFMK and its deformylated product *N*^1^-acetyl-5-methoxykynuramine (AMK) (Figure 4).

Both compounds were discovered after injection of melatonin into the *Cisterna cerebellomedullaris* (=*Cisterna magna*) of rats and were shown to be formed by enzymes present in rabbit brains [84]. As no substantial brain levels of 6-hydroxymelatonin were detected in that study, AFMK and AMK were believed to represent major catabolic products of melatonin in the CNS. However, the conclusion on absence of the 6-hydroxylation pathway in the CNS turned out to be precocious, since 6-hydroxymelatonin, surprisingly, disappeared by conjugation to 6-sulfatoxymelatonin in the brain, too [1,21]. Thereafter, several investigators tested the presence of AFMK and/or AMK in body fluids and some tissues, however, with a very moderate outcome [85,86,87,88]. In these studies, either of these substituted kynuramines were only detected in traces, sometimes only after melatonin injection. These findings strongly contrasted with the data of the original discovery. However, it seems important to remain aware that neither AFMK nor AMK have the primary function of acting as excretory products. Moreover, they do not seem to represent endocrine factors in the usual sense, although biological effects have been ascribed to both of them. To data, no high-affinity binding site has been detected for either of them. To better understand their roles, they should rather be perceived as locally produced metabolites with main actions at the sites of formation. Additionally, the conditions under which they are produced have to be taken into account.

AFMK can be generated by remarkably many processes [29,89,90], some of them by direct conversion of melatonin, others by chemical reactions of other melatonin metabolites (Figure 4). Direct formation from melatonin comprises various enzymatic, pseudoenzymatic, free radical-mediated and photochemical mechanisms summarized elsewhere [29]. A complete list of possible reactions would exceed the scope of this article, but a few reactions of relevance shall be mentioned.

The first enzyme that was shown to convert melatonin to AFMK was indoleamine 2,3-dioxygenase (IDO). However, it is important to keep in mind that this enzyme is not specific for melatonin, but rather converts various other indolic compounds, in particular, tryptophan, which is its main substrate. IDO is known to be upregulated by inflammatory signals, such as interferon-γ. The relationship to inflammation is even stronger in the case of myeloperoxidase, which also generates AFMK [91,92]. Under respective conditions, such as inflammation, it was concluded that about 35% of melatonin may enter the kynuric pathway [92]. AFMK formation from melatonin is also possible by other hemoproteins, such as some hemoperoxidases [29] and also cytochrome c [13]. The conversion by cytochrome c may receive more future attention with regard to the recent demonstration of melatonin biosynthesis in mitochondria [93]. Cytochrome c acts via a multi-step mechanism that involves AMIO (see above) and a further intermediate that is mentioned in literature as “2,3-dihydroxymelatonin” [13]. However, this name is, in fact, questionable, since such a compound is chemically inexistent. As already discussed above in the case of the so-called “2-hydroxymelatonin”, which rather tautomerizes almost completely to the respective indolinone, AMIO, this tautomerization has to be considered here even more and with imperative necessity, since a 2,3-dihydroxylated melatonin cannot exist because this would exceed the possible four bond numbers of carbon. Therefore, an additional hydroxylation at ring atom 3 is only possible at the indolinone tautomer, AMIO. As the 2,3-dienol is impossible, the term “2,3-dihydroxymelatonin” is strongly misleading and should be avoided. The intermediate may instead be called 3-hydroxy-AMIO or 3-OH-AMIO. Regardless of whether the preceding intermediate, AMIO, is formed by cytochrome c, or by two •OH or, in plants, by M2H, free AMIO can, of course, also be converted to 3-hydroxy-AMIO by two additional •OH (Figure 4). A look at the structural formulas immediately shows that the 3-hydoxy-AMIO molecule can easily undergo a spontaneous rearrangement to AFMK (Figure 4).

With regard to nonenzymatic AFMK formation, a few possibilities among many others shall be mentioned. If melatonin is 3-hydroxylated by two •OH to give cyclic 3-hydroxymelatonin, two further •OH can also generate AFMK (Figure 4) [29]. Another mode of formation was observed by using carbonate radicals (CO_3_•^−^) in a superoxide anion (O_2_•^−^)-generating system [90,94]. This shall be specifically mentioned because both of these free radicals are enhanced under conditions of mitochondrial dysfunction, e.g., in ischemia, and because increased CO_3_•^−^ formation is associated high levels of CO_2_ in mitochondria and in vascular underperfusion as well as at elevated peroxynitrite levels [95]. Finally, the formation of AFMK by singlet oxygen [O_2_(^1^Δ_g_)] deserves attention, as this reactive oxygen species is formed under the influence of UV light [96,97]. This represents a one-step addition mechanism, in which the oxygen directly combines with melatonin. The scavenging of singlet oxygen may be part of the photoprotective properties of melatonin that is of relevance to the human skin [98], to plants [22,66] and presumably to many other organisms [90,99]. Photocatalytic formation of AFMK was also observed using protoporphyrin IX as a photocatalyst [71,90,99], a compound of relevance to the high sensitivity of rodent Harderian glands to light and oxidative damage. Moreover, several extracts from slug integuments, from a brown alga and from the dinoflagellate *Lingulodinium* were shown to convert melatonin to AFMK under the influence of light, but the photocatalytically active components are only partially known [71,90,99].

In plants, AFMK formation has been rarely studied under natural conditions. A remarkable exception has been the case of the pontederiacean *Eichhornia crassipes* [100]. In plants kept under high natural irradiance and a natural temperature cycle, melatonin levels were considerably higher than under laboratory conditions and exhibited a diurnal rhythm with a peak around dusk. This rhythm was accompanied by a similar rhythm of AFMK, which amounted at peak to about 7% of melatonin concentration. Therefore, AFMK was, at least, a relevant product, whereas the proportion to other metabolites remained unknown, since AMIO was not yet identified as a major product at the time of that study. The mechanism of AFMK formation remains to be identified. An enzymatic contribution is possible, since an *IDO* gene was demonstrated to exist in rice and was also used as a transgene for lowering melatonin levels in tomatoes [101]. This shows that a melatonin-metabolizing IDO does exist in plants. Additional photochemical and other light-dependent reactions are also likely, since the high natural UV radiation should have caused formation of singlet oxygen and other reactive oxygen species. Singlet oxygen can be also formed in photosystem II [102]. Both photosystems can generate oxygen free radicals and hydrogen peroxide deriving thereof [103,104,105]. Apart from other effects, the reactive oxygen species can damage plastidial proteins, thereby further enhancing the formation rates of reactive oxygens. In cultured marine organisms such as *Lingulodinium*, this can be followed by the release of H_2_O_2_ into the seawater, which exhibits a strong increase over the photoperiod [106]. The light-induced oxidizing processes were shown to cause metabolization of other easily oxidizable aromates, such as kynurenine and 3-hydroxykynurenine to kynurenic and xanthurenic acids, respectively, with similar increases over the photoperiod and further enhancements upon extended light exposure [107,108].

AFMK may become of some future interest in plants, especially with regard to photochemical processes. Its formation was also shown in the chlorophycean *Chlorogonium elongatum*, a species at least phylogenetically related to plants, and in the unrelated phototroph, *Lingulodinium polyedrum* [109]. In these species, light was shown to increase melatonin catabolism with relatively high prevalence for AFMK formation, whereas in the respective media devoid of cells, mixtures of products were detected that were not found in the presence of the organisms. Similar observations were made in the ciliate *Paramecium bursaria*, which contained the chlorophycean symbiont *Chlorella*, but also in heterotrophs, such as *Paramecium caudatum* and the rotifer *Philodina acuticornis* [109]. AFMK formation was also detected in malaria parasites and this metabolite was reported to influence calcium levels and the cell cycle of *Plasmodium chabaudi* and *Plasmodium falciparum* [110].

The further metabolism of AFMK comprises well-known enzymatic processes and some less understood free radical-mediated reactions. Not surprisingly, AFMK interacts with the highly reactive •OH [111]. However, the scavenging efficiency is clearly below that of melatonin. Generally, AFMK is relatively inert compared to melatonin and also to its product AMK [29,90,112]. Therefore, protective effects by AFMK observed in biological systems [89] may well be attributable to AMK formed from it.

Although some studies detected only traces of AFMK in mammalian material [85,86,87,88], other data indicated a substantial relevance. AFMK was demonstrated in the human skin and cultured keratinocytes [25,63,98,113]. According to these studies, AFMK seems to be endogenously produced in the human skin, but its levels were shown to be enhanced by exogenous melatonin, including transdermal application [114], or by UV radiation [25]. In the skin, regulatory effects of AFMK were also demonstrated, which is remarkable because other biological actions mainly concerned antioxidative protection. AFMK was shown to increase the cutaneous formation of involucrin and keratins-10 and -14, effects that were also observed with melatonin [113]. This may indicate that AFMK mediates actions of melatonin in the skin. Under these perspectives, it seems that AFMK might be of particular importance to the skin as an organ exposed to visible and UV light as well as to other stress factors. It would be worth of investigating AFMK formation and actions in light-exposed tissues or cells of other organisms, too.

Most additional effects on expression of protective factors observed upon administration of AFMK may be interpreted as secondary consequences of its antioxidant capacity, including the formation of its metabolite AMK. An exception may have been found in a study on pancreatic cancer cells (PANC-1 cells), in which AFMK was reported to strongly affect the expression and distribution of heat shock proteins [115]. At the very low concentration of 10^−12^ M, a stimulation of HSP27 transfer from cytoplasm to the nucleus was observed, an upregulation of HSP70 and some more moderate effects on HSP90. In that study, authors tried to identify receptors involved in these actions and tested antagonists of MT_1_/MT_2_ and of 5-HT_3_ receptors. Inhibitions were especially observed with ketanserine. However, questions arise concerning the specificity of effects, because an entirely different kynuric compound, the amino acid l-kynurenine, exerted very similar effects at the same low concentration, and as the affinity of AFMK to melatonin receptors had previously been shown to be rather low [116,117]. The affinities of the secondary product AMK to melatonin receptors were likewise about 2 orders of magnitude lower than that of melatonin [118,119]. The possibility of signaling by AFMK remains to be clarified, but will presumably be decisive for judging the physiological relevance of this compound.

Another case of substantial mammalian AFMK levels has been reported to occur in the human CSF under brain inflammatory conditions [120]. In several patients with viral meningitis, the AFMK concentration exceeded 50 nM, which is by orders of magnitude higher than the nocturnal melatonin concentration in the blood plasma, which amounts to maximally 1 nM in young individuals, but less in middle-aged or older subjects. The findings are indicative of enhanced AFMK formation by inflammation, which may include oxidative stress and conversion of melatonin by myeloperoxidase. With regard to the inertness of AFMK, it may easily accumulate in the CSF.

The metabolism of AFMK is partially clarified. Although this has been suggested, the formation of AMK from AFMK by •OH is difficult to demonstrate. The considerably more reactive AMK is much more rapidly destroyed in •OH-generating systems than AFMK. In attempts of mimicking the •OH effects by using the ABTS [2,2’-azino-*bis*-(3-ethylbenzthiazoline-6-sulfonic acid)] cation radical, i.e., another electron-abstracting radical of lower reactivity but considerably longer lifetime, AFMK was transformed to various other, previously unknown products [121]. However, their biological relevance is still unknown. Therefore, this article will focus on the well-known and repeatedly studied metabolite AMK. The first-known reaction that leads to AMK is catalyzed by arylamine formamidases (Figure 4), a group of enzymes with rather low substrate specificity, which also accept AFMK as a substrate [29,122]. Other, again rather unspecific, but abundant enzymes, namely, hemoperoxidases, including catalase, were later convincingly shown to also catalyze the deformylation of AFMK, in a reaction via a carbamate intermediate that releases CO_2_ [123]. A third, direct photochemical reaction was later discovered, which consists in the liberation CO by sufficiently energetic photons, such as UV C or short-wave UV B [124]. While the hemoperoxidase mechanism should be possible in most cells from whatever taxon, the UV-dependent reaction should only be of interest in strongly UV-exposed organisms, such as high-altitude and desert plants.

The formation of AMK is of interest under various aspects. On the one hand, AMK has been shown to exert several effects on key enzymes of intercellular communication and stimulation of inflammatory responses. AMK was demonstrated to inhibit, as very low concentrations, neuronal NO synthase (nNOS) [125,126] and to downregulate the expression of inducible NO synthase (iNOS) [127]. Moreover, AMK is known since long to be a cyclooxygenase inhibitor more potent than acetylsalicylic acid [122]. Additionally, downregulation of cyclooxygenase 2 in macrophages was observed, however, using much higher concentrations [128]. Antioxidative protection including preservation of mitochondrial function was observed several times, especially, under conditions of high-grade inflammation [2,127,129].

**Figure 5:**
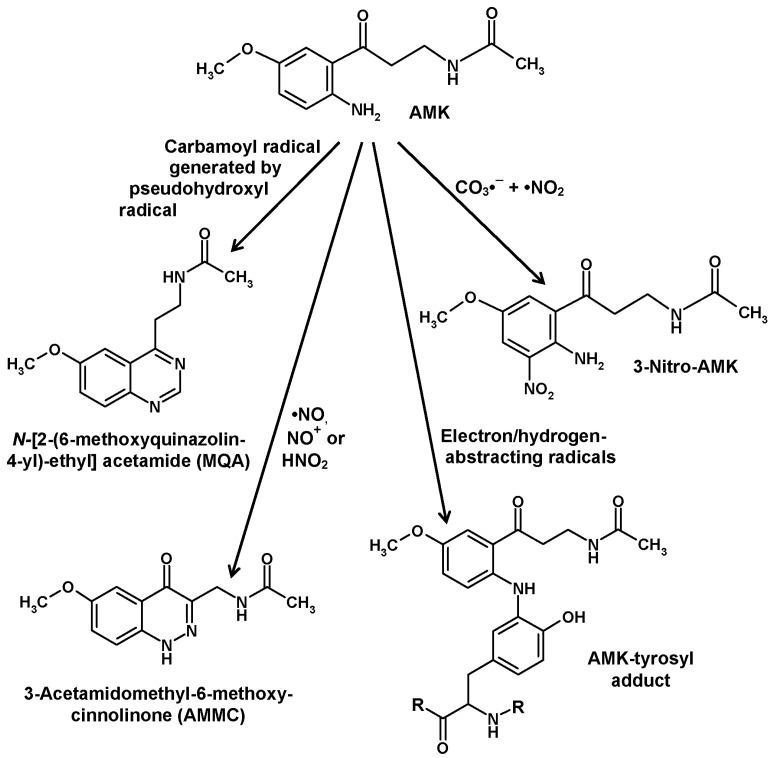
Various nonenzymatic reactions of *N*^1^-acetyl-5-methoxykynuramine (AMK). The AMK-tyrosyl adduct, as it was concluded to occur in some proteins, was deduced from interactions of AMK with the tyrosine side chain fragment, 4-ethylphenol [139]. Additionally, electron/hydrogen-abstracting radicals led to the formation of several chemically identified AMK dimers and oligomers [138] and to noncharacterized products formed by interaction with tryptophan [29].

The much higher chemical reactivity of AMK, compared to AFMK, has already been mentioned. This property, which leads to a rapid decay, represents an obstacle for detecting AMK in biological materials. Nevertheless, AMK was convincingly demonstrated in the human skin [63]. AMK efficiently scavenges •OH, CO_3_•^−^ and peroxyl radicals [90,95,112,130]. The direct products formed by these interactions have not been characterized because they turned out to be unstable and extremely short-lived [29]. However, scavenging of CO_3_•^−^ in combination with •NO_2_, which are generated by decomposition of the peroxynitrite-CO_2_ adduct, resulted in the formation of 3-nitro-AMK [131] (Figure 5). In the latter study, other previously unknown products of interactions of AMK with reactive nitrogen species were identified. AMK was shown to combine with •NO by forming a double-ring compound, 3-acetamidomethyl-6-methoxycinnolinone (AMMC) (Figure 5). Because of the newly formed ring, the adduct is stable [132,133], contrary to other nitroso-aromates including 1-nitrosomelatonin, which re-donates •NO, to compounds that form nitrosamines [132] or oxadiazoles and *o*-quinone diazides as shown for the structurally related kynurenine-derived metabolites 3-hydroxykynurenine and 3-hydroxyanthranilic acid [134]. AMMC was shown to by also produced from AMK by interactions with the reactive NO redox congeners, NO^+^ and HNO, the protonated form of NO^−^, in modifications of the reaction first observed with •NO [133].

Another compound that was easily formed from AMK by interaction with nitrogen compounds was identified as *N*-[2-(6-methoxyquinazolin-4-yl)-ethyl] acetamide (MQA) [131] (Figure 5). The possible reactions leading to this compound in biological material were not immediately clear, but a later study showed that this was catalyzed by carbamoylating reaction mixtures, especially by carbamoyl phosphate, a biological metabolite, in a reaction that was further enhanced by hydrogen peroxide and copper(II) [135]. These findings may reflect an aspect of copper toxicity and, perhaps, be indicative for a reactivity of AMK towards the so-called crypto- or pseudo-hydroxyl radical, which is assumed to be a copper- or cobalt-hydroperoxo complex that undergoes •OH-like reactions, but is not inhibited by classic •OH scavengers such as mannitol [130,135]. Detoxification of pseudo-hydroxyl radicals has been poorly studied, and also not by using melatonin. Notably, MQA formation was different from another well-known carbamoylation reaction by isocyanate, and it was also not produced by condensation of AFMK with ammonia, nor of AMK with formamide [130,135]. MQA has not been studied in mammalian tissues, but it was detected in relevant quantities, when yeast cells were incubated with AFMK [136]. As MQA is not a direct condensation product of AFMK, this should mean that AMK is formed from AFMK in *Saccharomyces*.

With regard to non-radical oxidative reactions, AMK proved to be one of the most potent scavengers of singlet oxygen [O_2_(^1^Δ_g_)] [97], which should have implications for all UV-exposed organisms and tissues. This property is of interest to dermatologists and, importantly, AMK was shown to be formed at substantial rates in the human skin [137]. AMK was over 150-fold more effective than the frequently used synthetic scavenger DABCO (diazabicyclo-(2,2,2)-octane) and still much better than imidazole (16-fold), *N*_α_-acetylhistidine (8-fold) and histidine (4-fold). Histidine is often applied in biological experiments, because it is usually considered to be only surpassed by polymeric singlet oxygen quenchers. However, melatonin is also more potent than histidine as a singlet oxygen scavenger, but AMK was still about 1.6 times more effective, whereas AFMK remained practically inert towards singlet oxygen under identical conditions [97].

The identification of oxidative metabolites formed from AMK had remained widely unsuccessful when using •OH, CO_3_•^−^, or singlet oxygen, because of rapid decay of the primary products. Therefore, studies were conducted using the less aggressive ABTS cation radical as an electron-abstracting agent. In these investigations, several products were detected and chemically characterized. Among them, three differently coupled dimers, a trimer and two tetramers were identified [138]. These products should not be regarded as being physiologically relevant, but they reflect reactions taking place at concentrations higher than in biological material. However, this study revealed the remarkably high reactivity of AMK-derived intermediates formed by electron abstraction in terms of attaching to other compounds, in particular, aromates. Subsequent studies using mixtures of AMK with tyrosine or tryptophan under oxidative conditions revealed that AMK-derived intermediates form adducts with these aromatic amino acids [29]. To avoid a high complexity of product mixtures, AMK was reacted with the tyrosine side-chain fragment, 4-ethylphenol, in the presence of ABTS cation radicals. In that investigation, the AMK adduct to the side chain was identified [139]. An adduct of AMK with free tyrosine seemed to be of less importance relative to the possibility that AMK might react with tyrosyl residues in proteins. The deduced AMK-tyrosyl adduct as it may occur in peptide chains is depicted in Figure 5.

The possibility that proteins might be AMKylated [29,139] has the potential of offering previously unexpected routes of inhibitory actions by AMK. In the focus of predictions were proteins that display easily accessible regulatory tyrosines at their cytosolic surface. Such tyrosines were assumed to be preferably AMKylated. This would be especially the case in tyrosine receptor kinases, and AMKylation might prevent tyrosine phosphorylation at those crucial regulatory sites, which would inhibit tyrosine receptor kinase-dependent cell proliferation [139]. Interestingly, a recent study on AMK in keratinocytes and melanoma cells demonstrated antiproliferative effects of AMK [137]. The most important finding in this context may be something that may have appeared to researchers as, in a sense, atypical or even questionable. AMK exerted these antiproliferative effects at very low concentrations between 10^−12^ and 10^−10^ M, but AMK never attained a half-maximal inhibition at higher doses [137]. This finding is reminiscent of results concerning the inhibition of nNOS [126]. Again, AMK exerted a demonstrable effect at very low concentrations, such as 20% inhibition at 10^−11^ M, but 50% inhibition required 70 µM. These concentration relationships appear incompatible with normal binding site kinetics. Apart from the fact that no high-affinity binding site for AMK is known in any organism, such findings may be interpreted in terms of effects obtained in two different concentration ranges. At the presumably low physiological AMK levels, protein AMKylation may allow a persistent inhibition that is limited by the available amounts of AMK, whereas other actions may be exerted at considerably higher levels by interference with other regulatory factors.

To date, studies on AMK have been mainly restricted to vertebrates or cell-free chemical systems. Moreover, AMK is not always easily accessible to experimental approaches because of its low concentrations. However, it is important to be aware that concentration may be mainly of importance for effects mediated via binding sites. Other effects concerning modification of reaction partners may instead be determined by rates of formation, especially if the adduct is rather stable. Because of its high reactivity, AMK can rapidly disappear even when it is generated at reasonable rates. Perhaps, it might be recommendable to not only determine AMK levels, but also to measure in the future amounts of its stable products, such as AMMC and MQA, which may allow conclusions on formation rates that are not deduced from AMK concentrations.

AMK may be of particular interest in the CNS, because of its NO-reducing actions, which contribute to the suppression of excitotoxicity and proinflammatory crosstalk with microglia and astrocytes. Moreover, its antioxidative, antiinflammatory and mitochondria-protecting properties may gain further attention. AMK in the skin seems to be an emerging field. However, it should not be overlooked that AMK may be of importance in all UV-exposed tissues and cells, including those of plants and other phototrophs. This might include its interactions with reactive nitrogen species. In plants, •NO is meanwhile known to be formed by four NO synthases [140], to exert effects in mitochondria [141] and in chloroplasts [142], to be involved in stomatal closure, plants immunity responses and cell death [143]. A compound like AMK that multiply interacts with •NO and also detoxifies singlet oxygen and various free radicals should become of interest to the botanical field, especially as its precursor, AFMK, has already been demonstrated in plants.

## 6. Conclusions

Among the melatonin-catabolizing pathways, enzymatic and nonenzymatic pathways may be differently judged in terms of taxon and site specificity. While the enzymatic pathways can be strongly specific for certain groups of organisms and may also vary from tissue to tissue, nonenzymatic processes may occur in almost all species and cell types. However, this does not mean that the nonenzymatic reactions take place everywhere at similar rates or have the same biological relevance. Elevated rates of nonenzymatic melatonin catabolism can be expected in species and tissues exposed to potentially noxious influences, such as high formation rates of free radicals and singlet oxygen. This should be so in cases of UV exposure, especially, in plants living in unshaded subtropical or tropical and high-altitude habitats. In plants, free radical-mediated melatonin catabolism should also be caused by organelles forming reactive oxygen species at high rates, in particular, chloroplasts, but also mitochondria. In the chloroplasts, visible light absorbed by light-harvesting antenna complexes may already cause formation of free radicals and singlet oxygen by the photosystems, processes that are strongly enhanced as soon as components of the photosystems become damaged in the course of extended light exposure. UV-induced melatonin catabolism is presumably also of relevance to the skin of humans and those vertebrates that are poorly protected by fur or plumage, either in parts of the body or on their entire surface. Same assumptions may be made for strongly light-exposed invertebrates, but this has never been investigated directly. To date, only the destruction of melatonin and formation of AFMK has been studied using extracts of slug integuments exposed to visible light [99], conditions that may be far from physiological.

In vertebrates, nonenzymatic melatonin catabolism can be assumed to be elevated at sites of inflammation, because of the increased rates of superoxide and HOCl release by neutrophils and macrophages. In all these cases, enhanced nonenzymatic melatonin hydroxylation, e.g., to cyclic 3-hydroxymelatonin, and generation of AFMK and AMK can be expected. If AMK is formed under inflammatory conditions, the concomitant release of •NO should lead to AMMC. In plants, too little is yet known about AMK.

The enzymatic routes of melatonin catabolism, which are of much higher quantitative importance, have turned out to be strongly taxon-specific and, at least, in vertebrates, partially site-specific. In vertebrates, the typical route of melatonin elimination is known since long to be hepatic 6-hydroxylation followed by sulfation, which allows efficient excretion. However, this route is not that much exclusive to liver as frequently assumed, since it takes also place in the CNS [1]. This route of brain metabolism may be of higher importance than previously believed, because the amounts of melatonin released directly to the CSF via the pineal recess [144] have now been considered as being much more important than thought before [145,146]. The kynuric pathway, once believed to represent a major route of melatonin catabolism in the brain [84], now appears to be mainly of relevance under inflammatory conditions. However, the kynuric pathway of melatonin has now gained relevance and attention in the human skin [63,98,113,137].

Melatonin catabolism in plants is obviously entirely different from a quantitative point of view. The main metabolite is clearly formed by M2H, which hydroxylates melatonin at ring atom 2 to give a metabolite that tautomerizes to AMIO. The extremely high amounts of this substance that exceed by far those of the parent compound may, on the one hand, reflect its chemical inertness, but, on the other hand, indicate a plant physiological role, which may be sought in stress resistance, as recently suggested [41,42]. The only demonstration of enzymatic 3-hydroxylation of melatonin, by M3H, is still restricted to bacteria and plants carrying bacterial transgenes [3].

An entirely different situation is found in dinoflagellates, in which melatonin is preferably metabolized to 5-MT. This is especially of importance under cyst-inducing conditions, which first lead to enormous upregulations of melatonin, followed by formation of 5-MT, which represents the direct inducer of encystment [66,76,77,78]. In these organisms, 5-MT is metabolized to 5-methoxyindole 3-acetaldehyde and further to 5-MIAA, to a minor extent, also to 5-ML. The major final product, 5-MIAA, is directly released to the slightly alkaline seawater, a convenient method for these cells to eliminate the end product without energy consumption. Formation of 5-MT is also of importance for eliminating melatonin in retinas of non-mammalian vertebrates and in pineal glands. This route is, insofar, also taxon- or site-specific, but the quantitative relevance is not comparable to the almost exclusive role in dinoflagellates nor to hepatic melatonin catabolism in vertebrates.
